# Chemoselective reduction of aldehydes by ruthenium trichloride and resin-bound formates

**DOI:** 10.3762/bjoc.4.53

**Published:** 2008-12-19

**Authors:** Basudeb Basu, Bablee Mandal, Sajal Das, Pralay Das, Ashis K Nanda

**Affiliations:** 1 Department of Chemistry, University of North Bengal, Darjeeling 734 013, India

**Keywords:** Amberlite^®^ resins, chemoselectivity, hydrogen transfer, reduction of carbonyl group, ruthenium chloride

## Abstract

A simple, chemoselective transfer hydrogenation of aryl aldehydes with the aid of Amberlite^®^ resin formate (ARF), a stable H-donor, in the presence of catalytic ruthenium trichloride is described. Aromatic aldehydes and 1,2-diketones are reduced efficiently and selectively, while aryl ketones remain unchanged. Several other potentially reducible groups attached to the aromatic moiety are unaffected.

## Introduction

Reduction of carbonyl functionality by transition metal-catalyzed transfer hydrogenation (CTH) with the aid of a suitable hydrogen donor is a valuable synthetic tool and has proved to be a viable alternative to hydrogenation using molecular hydrogen [1–3]. Since hydrogenation using molecular hydrogen is associated with risks and often requires high pressure apparatus, the alternative technique, CTH, has been employed in many laboratories. In transfer hydrogenation, several organic molecules such as hydrocarbons [4], primary and secondary alcohols [5–6], and formic acid and its salts [7–11] have been used as the hydrogen source. Besides the use of Rh, Ir, Ni and Pd metals in CTH, carbonyl reduction using the combination of Ru(II)-ligand complexes and propan-2-ol in the presence of a base is a widely used method in modern organic synthesis [5]. The ability of DMA (*N,N*-dimethylacetamide) or DMF (*N,N*-dimethylformamide) solutions of RuCl_3_ to catalyze hydrogenation of simple olefins has long been recognized [12–13]. However, only recently, James and coworkers demonstrated the first example of the use of a simple, phosphine-free, RuCl_3_-DMA catalytic system in H_2_-hydrogenation of dimethyl ester of protoporphyrin IX to the mesoporphyrin analogue [14]. Catalytic activity of styrene-divinyl benzene copolymer-bound Ru(III)-EDTA complex was also studied in H_2_-hydrogenation of alkenes [15]. In the case of CTH, although there are some reports on the use of well-defined ortho-metalated and cyclo-metalated Ru(III) complexes and propan-2-ol (as the hydrogen source) in the presence of a base [16–20], there has been no systematic investigation on the use of RuCl_3_ in CTH of various organic functional groups.

**Scheme 1:**
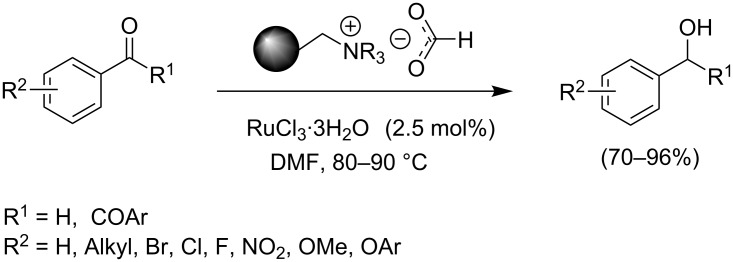
RuCl_3_-catalyzed transfer hydrogenation of aryl aldehydes.

Reagents immobilized on polymer supports have emerged as potentially attractive tools in terms of clean and green reactions, ease of separation of the products and reusability [21–22]. We have recently demonstrated that poly-ionic resin formate can act as a stable and potent hydride source in Pd-catalyzed transfer hydrogenation of functionalized alkenes, imines, nitroarenes and 1,2-diketones [23–24]. Danks et al. also carried out reduction of alkyl cinnamates using polymer supported formate and catalytic RhCl(PPh_3_)_3_ (2.5 mol%) under microwave irradiation [25]. Pd-catalyzed transfer hydrogenation of nitroarenes using recyclable polymer-supported formate has been investigated by Abiraj et al [26]. Neither of these conditions were, however, effective in reducing aryl ketones. Since aryl alcohols are important compounds, we became interested to look at the ability of Ru(III) salts in the CTH of aryl ketones using the poly-ionic resin formate. Our studies reported herein constitute an efficient method for chemoselective transfer hydrogenation of aryl aldehydes with the aid of resin-supported formate in the presence of catalytic (2.5 mol%) amount of commercially available RuCl_3_·3H_2_O in DMF or DMA solution (Scheme 1).

## Results and Discussion

In order to optimize the reaction conditions and to find the minimum catalyst requirement, we began our studies with *p*-anisaldehyde using Amberlite^®^ resin formate (ARF) (0.5 g/mmol of the substrate) as the reducing source. A variety of conditions were investigated, which are summarized in Table 1. Taking 5 mol% of RuCl_3_·3H_2_O in DMF and stirring the reaction mixture at 100 °C for 10 h afforded the desired alcohol in 81% yield (Table 1, entry 1). A similar yield of the alcohol was also obtained on carrying out the reaction in presence of 2.5 mol% of RuCl_3_·3H_2_O at 80 °C for 8 h (Table 1, entry 6). Further lowering of the amount of RuCl_3_·3H_2_O or the reaction temperature, however, led to reduced yield (Table 1, entries 7–9). In order to compare the efficiency of the catalytic combination of the reductant (ARF/RuCl_3_·3H_2_O), we carried out the CTH using a well-defined Ru(II) complex [Dichloro(*p*-cymene)ruthenium(II)] dimer; (2 mol%) under similar conditions and indeed a comparable result was observed (Table 1, entry 11). On the basis of this comparison, it may be presumed that the Ru(III) salt might undergo *in situ* reduction to Ru(II), which then catalyzes the hydrogenation of the aldehydes.

**Table 1:** Optimization of the reaction conditions.^a^

Entry	Catalyst (mol%)	Temp (°C)	Solvent	t (h)	Yield (%)^b^

1	5.0	100	DMF/DMA	10	81
2	5.0	80	DMF	10	84
3	5.0	60	DMF	10	21
4	2.5	100	DMF/DMA	10	80
5	2.5	80	DMF	10	81
6	2.5	80	DMF	8	83
7	1.0	80	DMA	10	n.d.^c^
8	2.5	60	DMF	12	29
9	2.0	100	DMA	12	41
10^d^	5.0	100	DMF	12	n.d.^c^
11^e^	2.0	100	DMF	8	80
12^f^	2.5	80	DMF	8	63

^a^*p*-Anisaldehyde (1 mmol) and ARF (0.5 g mmol^−1^) in DMF or DMA under nitrogen. ^b^Yield of isolated product. ^c^Not detected by HPLC. ^d^*p*-Methoxyacetophenone (1 mmol) and ARF (0.5 g mmol^−1^). ^e^Dichloro(*p*-cymene)-ruthenium(II) dimer used instead of RuCl_3_·3H_2_O. ^f^HCOOK (2 equiv) was used as the reducing source instead of ARF.

The ARF was prepared from commercially available Amberlite^®^ resin (chloride form) by exchanging the anion (chloride) with formic acid following our procedure [24]. A wide range of aryl aldehydes were subjected to reduction under the optimized conditions. Aryl aldehydes substituted with various electron withdrawing and donating groups did not seem to influence the reduction rate as revealed by the similarity of the results and all gave the corresponding alcohols in high yields (Table 2). Several potentially reducible groups such as halogens, nitro etc. were not affected under the reaction conditions (Table 2, entries 6–11, 17). Aliphatic aldehydes (Table 2, entries 14, 15) were also reduced to corresponding alcohols efficiently. Furthermore, the presence of ortho-substituents did not hinder the rate of the reduction as manifested from the reaction conditions (Table 2, entries 6, 9). Hetero-aryl aldehydes were also reduced to corresponding alcohols efficiently (Table 2, entries 16, 17). Surprisingly, aryl ketones were not reduced under similar conditions despite a great deal of variation in experimental conditions (addition of bases, phosphine ligands and application of higher temperatures up to 120 °C). The selectivity between aryl aldehyde and aryl ketone might offer a distinct advantage when both the functional groups are present. Accordingly, we applied the protocol to a mixture of an aryl aldehyde and aryl ketone (1 mmol each). After conducting the reaction at 85 °C for 8 h, the aryl ketone was recovered almost quantitatively along with the reduced product of the aldehyde (Table 2, entry 18). Distinct advantages of cleaner reaction and easy isolation of the product are notable features when comparing the application of heterogeneous ARF and a simple formate salt (herein potassium formate) in homogeneous phase (Table 1, entry 12). The resin beads obtained after filtration from the reaction mixture could be reused for further hydrogenation reactions after washing with methanol and recharging with formic acid.

**Table 2:** Reduction of aryl aldehydes using resin-supported formate and catalytic RuCl_3_·3H_2_O.

Entry	Substrate	Product	Conditions^a^ Temp / Time	Yield (%)^b^

1	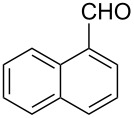	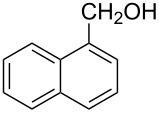	80 °C / 8 h	91
2	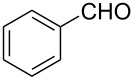	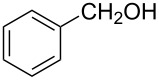	80 °C / 8 h	74
3	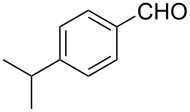	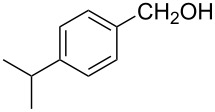	85 °C / 9 h	83
4	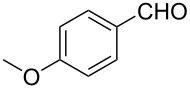	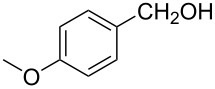	85 °C / 8 h	83
5	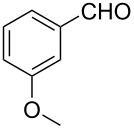	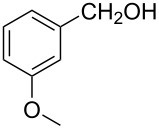	80 °C / 8 h	70
6	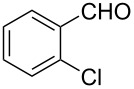	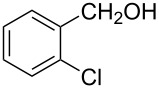	90 °C / 7 h	78
7	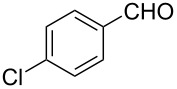	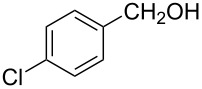	85 °C / 8 h	70
8	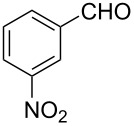	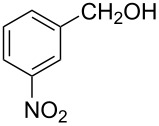	80 °C / 8 h	96
9	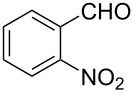	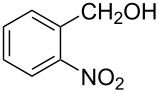	90 °C / 9 h	84
10	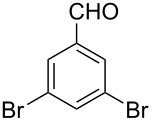	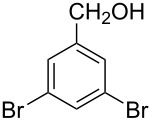	85 °C / 8 h	72
11	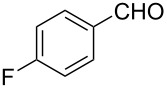	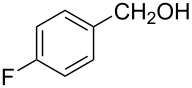	90 °C / 8 h	79
12	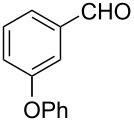	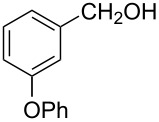	90 °C / 8 h	81
13	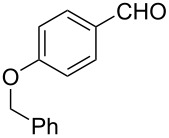	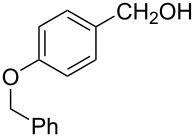	90 °C / 8 h	86
14	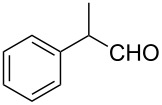	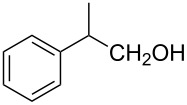	80 °C / 8 h	79
15	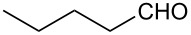	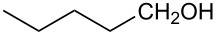	80 °C / 12 h	94
16	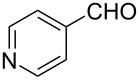	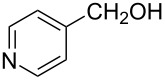	80 °C / 8 h	83
17	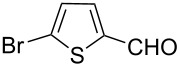	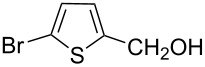	80 °C / 8 h	76
18	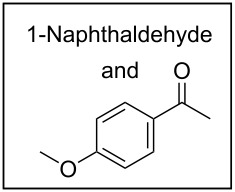	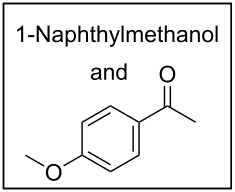	85 °C / 8 h	89^c^

^a^Aldehyde/ARF/RuCl_3_·3H_2_O (1 mmol:500 mg:0.025 mmol) in 2 ml DMF (or DMA). ^b^Isolated yields are average of two runs and alcohols are characterised by spectral data. ^c^Nearly quantitative recovery of ketone.

The reaction conditions appear to be mild and base-free, and give high yields of the corresponding alcohols and free of any by-product. Of interest is that, although the use of base co-catalysts for metal complex catalyzed hydrogen transfer is common [27–30], the present reaction conditions without any base preclude possibilities of unwanted reactions of aryl aldehydes, e.g. Cannizzaro reaction.

**Scheme 2:**
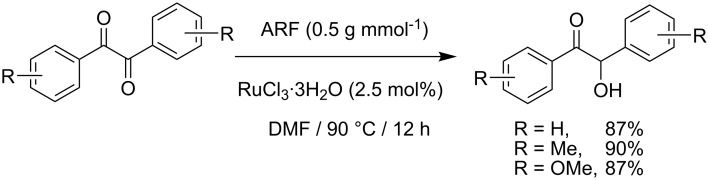
Reduction of 1,2-diketones.

To broaden the scope of the catalytic system, we tested CTH of 1,2-diketones under similar conditions (Scheme 2). Whereas aryl ketones were not reduced under the conditions, reduction of benzil to benzoin proceeded smoothly in good to excellent yields. Until now, various procedures [31–32] including Lewis acid-mediated conditions [32] have been developed for the reduction of 1,2-dicarbonyl compounds to yield the α-hydroxy ketones without over reduction to diols. The direct use of catalytic RuCl_3_·3H_2_O in combination with ARF under neutral conditions could be of interest. Diketones with other substituents also worked efficiently and the results are presented in Scheme 2.

## Conclusion

In summary, this system i.e. ARF–RuCl_3_–DMF (or DMA), constitutes an efficient and selective reductant for reduction of aryl aldehydes and 1,2-diketones to aryl alcohols and α-hydroxy ketones respectively under mild, base-free and phosphine- or any ligand-free conditions. It is observed that aryl ketones and several other potentially reducible functionalities remain unchanged under the reaction conditions. The catalytic system should find further applications since no specially designed chelating ligand-based Ru-complexes are required and the resin-supported H-source (ARF) is easy to prepare and can be stored at room temperature for several months without special precautions.

## Experimental

*A representative procedure for RuCl*_3_·*3H*_2_*O-catalyzed transfer hydrogenation of aryl aldehyde using ARF*: 1-Naphthaldehyde (156 mg, 1 mmol), ARF (500 mg), RuCl_3_·3H_2_O (6.5 mg, 2.5 mol%) and DMF (2 mL) were placed in a screw-capped tube and heated in an oil bath at 80 °C for 8 h. The mixture was cooled, diluted with water (4 mL) and then the resins were filtered off by passing through a cotton bed. The filtrate was diluted with water, extracted with ether (2 × 10 mL) and the combined organic layers were washed with brine and dried over Na_2_SO_4_. Removal of the solvent afforded an oil, which was purified through a small pad of silica gel (mesh size 60–120) using ethyl acetate/light petroleum (1:4) to give 1-naphthylmethanol as a colorless solid (144 mg, 91% yield); mp 59–60 °C (Lit. [33] mp 60–62 °C), FT-IR (Nujol): ν_max_ 3317, 2877, 1596, 1512 cm^−1^; ^1^H NMR (CDCl_3_, 300 MHz): δ 8.07–8.04 (m, 1H), 7.86–7.76 (m, 2H), 7.51–7.31 (m, 4H), 5.07 (s, 2H), 2.14 (br s, 1H); ^13^C NMR (CDCl_3_, 75 MHz): δ 136.3, 133.8, 131.2, 128.7, 128.6, 126.3, 125.9, 125.4, 125.3, 123.7, 63.6.

*Representative procedure for RuCl*_3_·*3H*_2_*O-catalyzed transfer hydrogenation of aryl aldehyde using HCOOK*: 1-Naphthaldehyde (156 mg, 1 mmol), HCOOK (168 mg, 2 mmol), RuCl_3_·3H_2_O (6.5 mg, 2.5 mol%) and DMF (2 mL) were placed in a screw-capped tube and heated in an oil bath at 80 °C for 8 h. The mixture was cooled and diluted with water (4 mL) followed by extraction with ether (2 × 10 mL). The combined organic extracts were then washed with brine and dried over anhydrous Na_2_SO_4_. Removal of the solvent afforded an oil, which after purification by column chromatography through a small pad of silica gel (mesh size 60–120) using ethyl acetate/light petroleum (1:4) gave 1-naphthylmethanol as a colorless solid (100 mg, 63% yield); mp 57–59 °C.

## Supporting Information

Supporting information features general experimental procedures and IR, ^1^H and ^13^C NMR spectral data for alcohols (Table 2, entries 3, 5, 6, 8–14, 16, 17) and HRMS data for alcohols (Table 2, entries 3, 12, 13, 14).

File 1 General experimental procedure

File 2 Spectral data of some selected alcohols
