# Supplementary material for: Chemoselective reduction of aldehydes by ruthenium trichloride and resin-bound formates
Source: Beilstein J Org Chem. 2008 Dec 19;4:53. doi: 10.3762/bjoc.4.53 (PMC2633666; doi:10.3762/bjoc.4.53)
Supplement: File 1 — General experimental procedure [file Beilstein_J_Org_Chem-04-53-s001.pdf]

Supporting Information for

Chemoselective reduction of aldehydes by ruthenium trichloride and resin-bound formates

Basudeb Basu*, Bablee Mandal, Sajal Das, Pralay Das and Ashis K. Nanda

Address: Department of Chemistry, University of North Bengal, Darjeeling 734 013, India.

Email: Basudeb Basu* - basu_nbu@hotmail.com

General Information

All the reductions were carried out under nitrogen. DMF was freshly distilled. Amberlite® IRA 900 (Chloride form) was purchased from Acros Organics, Belgium and used as received. All other reagents were used as received without any further purification. Column chromatography: silica (60–200 μm) (SRL, India), TLC on Merck plates coated with silica gel 60, F254. Solutions for NMR spectra of the organic compounds were recorded on Bruker AV 300 and 400 spectrometers using TMS as the internal standard. High-resolution mass spectra were obtained using an Qtof Micro YA263 instrument. FT-IR spectra were recorded on a Shimadzu-8300 spectrophotometer using KBr pellets.

General Procedure

The aldehyde (1 mmol), ARF (500 mg), $RuCl_3{\cdot}3H_2O$ (6.5 mg, 2.5 mol%) and DMF (2 mL) were placed in a screw-capped tube and heated in a preheated oil bath at 80–90 °C for 7–12 h. The mixture was cooled, diluted with water (4 mL) and then the resins were filtered off by passing through a cotton bed. The filtrate was diluted with water, extracted with ether (2 x 10 mL) and the combined organic layers were washed with brine and dried over Na_2SO_4. Removal of the solvent afforded an oil, which was purified through a small path of silica gel (mesh size 60–120) using ethyl acetate/light petroleum as the eluent to afford the pure alcohol.
