# Supplementary material for: Chemoselective reduction of aldehydes by ruthenium trichloride and resin-bound formates
Source: Beilstein J Org Chem. 2008 Dec 19;4:53. doi: 10.3762/bjoc.4.53 (PMC2633666; doi:10.3762/bjoc.4.53)
Supplement: File 2 — Spectral data of some selected alcohols [file Beilstein_J_Org_Chem-04-53-s002.pdf]

Supporting Information for

Chemoselective reduction of aldehydes by ruthenium trichloride and resin-bound formates

Basudeb Basu*, Bablee Mandal, Sajal Das, Pralay Das and Ashis K. Nanda

Address: Department of Chemistry, University of North Bengal, Darjeeling 734 013, India.

Email: Basudeb Basu* - basu_nbu@hotmail.com

Spectral data of some selected alcohols

Table 2, Entry 3: (4-Isopropylphenyl)methanol [1]

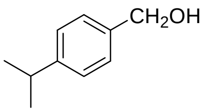

IR (neat) ν_{max}: 3334, 2960, 2871, 1514, 1215 cm^{-1}. ^{1}H NMR ($CDCl_3$, 400 MHz) δ = 7.29 (2H, d, *J* = 8.0 Hz), 7.23 (2H, d, *J* = 8.0 Hz), 4.65 (2H, s), 2.90 (1H, septet, *J* = 6.9 Hz), 1.66 (1H, br s), 1.25 (6H, d, *J* = 6.9 Hz). ^{13}C NMR ($CDCl_3$, 100 MHz) δ = 147.5, 137.3, 126.2, 125.6, 64.3, 32.9, 22.9. HRMS: Calcd for $C_{10}H_{14}ONa$: $[M+Na]^+$, 173.0943; found: 173.0946.

Table 2, Entry 5: (3-Methoxyphenyl)methanol [2]

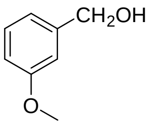

IR (neat) ν_{max}: 3332, 2912, 2837, 1587, 1265 cm^{-1}. ^{1}H NMR ($CDCl_3$, 300 MHz) δ = 7.25 (1H, t, *J* = 7.5 Hz), 6.92–6.81 (3H, m), 4.62 (2H, s), 3.79 (3H, s), 2.41 (1H, br s). ^{13}C NMR ($CDCl_3$, 75 MHz) δ = 159.7, 142.5, 129.5, 119.0, 113.1, 112.2, 65.0, 55.1.

Table 2, Entry 6: (2-Chlorophenyl)methanol [3]

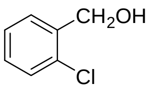

mp = 73–74 °C (Lit. 74 °C). IR (Nujol) ν_{max}: 3201, 2854, 1458, 1034 cm^{-1}. ^{1}H NMR ($CDCl_3$, 300 MHz) δ = 7.46 (1H, dd, *J* = 7.5 & 2.1 Hz), 7.35 (1H, dd, *J* = 7.2 & 1.8 Hz), 7.28 (1H, ddd, *J* = 7.5, 7.2 & 1.8 Hz), 7.23 (1H, ddd, *J* = 7.2, 7.2 & 2.1 Hz), 4.75 (2H, s), 2.31 (1H, s). ^{13}C NMR ($CDCl_3$, 75 MHz) δ = 138.1, 132.6, 129.3, 128.8, 128.6, 127.0, 62.7.

Table 2, Entry 8: (3-Nitrophenyl)methanol [4]

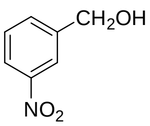

IR (neat) ν_{max}: 3360, 2874, 1528, 1350, 1046 cm^{-1}. ^{1}H NMR ($CDCl_3$, 300 MHz) δ = 8.18 (1H, s), 8.08 (1H, dd, *J* = 7.8 & 1.8 Hz), 7.64 (1H, d, *J* = 7.5 Hz), 7.47 (1H, t, *J* = 7.8 Hz), 4.76 (2H, s), 2.19 (1H, br s). ^{13}C NMR ($CDCl_3$, 75 MHz) δ = 148.3, 142.8, 132.6, 129.4, 122.4, 121.4, 63.9.

Table 2, Entry 9: (2-Nitrophenyl)methanol [5]

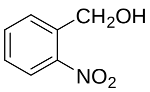

IR (neat) ν_{max}: 3364, 2877, 1532, 1349, 1047 cm^{-1}. ^{1}H NMR ($CDCl_3$, 300 MHz) δ = 8.09 (1H, d, *J* = 8.1 Hz), 7.74 (1H, d, *J* = 7.5 Hz), 7.67 (1H, dd, *J* = 8.1 & 7.8 Hz), 7.47 (1H, dd, *J* = 7.8 & 7.6 Hz), 4.97 (2H, s), 2.61 (1H, br s). ^{13}C NMR ($CDCl_3$, 75 MHz) δ = 136.8, 134.2, 129.9, 128.5, 125.0, 62.5.

Table 2, Entry 10: (3,5-Dibromophenyl)methanol [6]

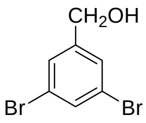

mp = 98 °C. IR (Nujol) ν_{max}: 3309, 2925, 2854, 1556, 1033 cm^{-1}. ^{1}H NMR ($CDCl_3$, 400 MHz) δ = 7.56 (1H, s), 7.42 (2H, s), 4.62 (2H, s), 2.15 (1H, s). ^{13}C NMR ($CDCl_3$, 100 MHz) δ = 144.6, 133.0, 128.4, 123.0, 63.6.

Table 2, Entry 11: (4-Fluorophenyl)methanol [7]

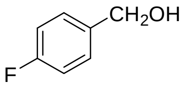

IR (neat) ν_{max}: 3332, 2879, 1514, 1417, 1222 cm^{-1}. ^{1}H NMR ($CDCl_3$, 300 MHz) δ = 7.31–7.23 (2H, m), 7.05–6.96 (2H, m), 4.58 (2H, s), 2.50 (1H, s). ^{13}C NMR ($CDCl_3$, 75 MHz) δ = 163.9, 160.6, 136.6, 136.5, 128.8, 128.7, 115.5, 115.2, 64.4.

Table 2, Entry 12: (3-Phenoxyphenyl)methanol

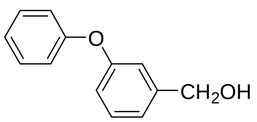

IR (neat) ν_{max}: 3319, 3039, 2871, 1583, 1446, 1215 cm^{-1}. ^{1}H NMR ($CDCl_3$, 400 MHz) δ = 7.36–7.29 (3H, m), 7.13–7.07 (2H, m), 7.02–7.00 (3H, m), 6.94–6.91 (1H, m), 4.62 (2H, s), 2.20 (1H, br s). ^{13}C NMR ($CDCl_3$, 100 MHz) δ = 157.4, 156.9, 142.9, 129.7, 129.6, 123.2, 121.5, 118.9, 117.7, 116.3, 64.5. HRMS: Calcd for $C_{13}H_{12}O_2Na$: [M+Na]$^+$, 223.0735; found: 223.0739.

Table 2, Entry 13: [4-(Benzyloxy)phenyl]methanol

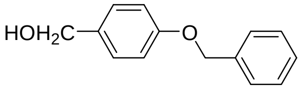

mp = 90 °C. IR (neat) ν_{max}: 3379, 2923, 2856, 1508, 1456, 1244 cm^{-1}. ^{1}H NMR ($CDCl_3$, 400 MHz) δ = 7.34–7.27 (7H, m), 6.97–6.95(2H, m), 5.07 (2H, s), 4.60 (2H, s). ^{13}C NMR ($CDCl_3$, 75 MHz) δ = 158.3, 136.9, 133.4, 128.6, 127.9, 127.4, 114.9, 70.0, 64.8. HRMS: Calcd for $C_{14}H_{14}O_2Na$: [M+Na]$^+$, 237.0892; found: 237.0899.

Table 2, Entry 14: 2-Phenylpropan-1-ol

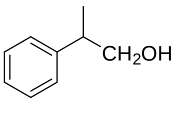

IR (neat) ν_{max}: 3350, 3010, 2950, 1600, 1500 cm^{-1}. ^{1}H NMR ($CDCl_3$, 400 MHz) δ = 7.38–7.34 (2H, m), 7.28–7.24 (3H, m), 3.73 (2H, s), 2.97 (1H, q, J = 6.96 Hz), 1.31 (3H, d, J = 7.08 Hz). ^{13}C NMR ($CDCl_3$, 75 MHz) δ = 143.8, 128.6, 127.5, 126.7, 68.7, 42.4, 17.6. HRMS: Calcd for $C_9H_{12}ONa$: [M+Na]$^+$, 159.0786; found: 159.0789.

Table 2, Entry 16: (Pyridin-4-yl)methanol [8]

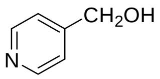

mp = 58 °C (Lit. 57–60 °C). IR (Nujol) ν_{max}: 3125, 2854, 1605, 1042 cm^{-1}. ^{1}H NMR ($CDCl_3$, 300 MHz) δ = 8.33 (2H, dd, *J* = 4.5 & 1.8 Hz), 7.21 (2H, dd, *J* = 4.5 & 1.8 Hz), 4.64 (2H, s), 4.20 (1H, br s). ^{13}C NMR ($CDCl_3$, 75 MHz) δ = 151.5, 149.0, 121.3, 62.8.

Table 2, Entry 17: (5-bromothiophen-2-yl)methanol [9]

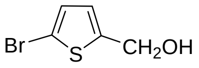

IR (neat) ν_{max}: 3321, 2927, 2869, 1014 cm^{-1}. ^{1}H NMR ($CDCl_3$, 400 MHz) δ = 6.90 (1H, d, *J* = 3.68 Hz), 6.72 (1H, d, *J* = 3.64 Hz), 4.69 (2H, s), 2.42 (1H, br s). ^{13}C NMR ($CDCl_3$, 100 MHz) δ = 145.7, 129.6, 125.6, 112.2, 60.0.

References

1. Van Tamelen, E. E.; Gladysz, J. A. *J. Am. Chem. Soc.* **1974,** *96,* 5290–5291. doi:10.1021/ja00823a065
2. Moiseev, D. V.; James, B. R.; Hu, T. Q. *Inorg. Chem.* **2006,** *45,* 10338–10346. doi:10.1021/ic0613560
3. Lane, C. F.; Myatt, H. L.; Daniels, J.; Hopps, H. B. *J. Org. Chem.* **1974,** *39,* 3052–3054. doi:10.1021/jo00934a028
4. Thorp, L.; Wildman, E. A. *J. Am. Chem. Soc.* **1915,** *37,* 372–377. doi:10.1021/ja02271a013
5. Raghuvanshi, R. S.; Singh, K. N. *Synth. Commun.* **2007,** *37,* 1371–1374. doi:10.1080/00397910701228604
6. El Bakkari, M.; Vincent, J.-M. *Org. Lett.* **2004,** *6,* 2765–2767. doi:10.1021/ol048994b

7. Kabalka, G. W.; Malladi, R. R. *Chem. Commun.* **2000,** 2191. doi:10.1039/b007190k
8. Boekelheide, V.; Linn, W. J. *J. Am. Chem. Soc.* **1954,** *76,* 1286–1291. doi:10.1021/ja01634a026
9. Vu, T.-T.-H.; Kumbhar, P. S.; Figueras, F. *Adv. Synth. Catal.* **2003,** *345*, 493–496. doi:10.1002/adsc.200390056
